# Magnetic Needle Steering in Soft Phantom Tissue

**DOI:** 10.1038/s41598-020-59275-x

**Published:** 2020-02-12

**Authors:** Mahdi Ilami, Reza James Ahmed, Alex Petras, Borhan Beigzadeh, Hamid Marvi

**Affiliations:** Arizona State University, School for Engineering of Matter Transport and Energy (SEMTE), Tempe, 85287 USA

**Keywords:** Surgery, Biomedical engineering

## Abstract

Needle steering is a technology for guiding needles around sensitive internal obstacles in minimally invasive surgery. Traditional techniques apply rotation at the base of a needle with an asymmetric tip, enabling steering through the redirection of radial forces. Magnetic steering of catheters and continuum manipulators is another technology that allows steering of a shaft in the body. Both of these techniques rely on mechanical or manual shaft advancement methods. Needle steering has not achieved widespread clinical use due to several limitations: 1- buckling and compression effects in the shaft and needle rotation cause excessive tissue damage; 2- torsion effects on the shaft and needle deflection at tissue boundaries lead to difficulty in control; and 3- restricted radius of curvature results in limited workspace. Magnetically steered catheters and continuum manipulators also suffer from limited curvature and the possibility of buckling. This paper proposes a novel needle steering method empowered by electromagnetic actuation that overcomes all of the aforementioned limitations, making it a promising option for further study toward healthcare applications.

## Introduction

Needles are among the least invasive surgical tools available to doctors and surgeons. The wound caused by a needle is easily and quickly repaired by the body and is, therefore, the preferred method of administering or drawing liquids to or from the body. Inflexible needles can only reach a target just under the skin, and not one protected by bone or sensitive tissues. However, needles with flexible, long shafts can be steered around these internal anatomies. The benefits of the ability to tightly steer around sensitive or protective internal obstacles can be seen in several medical applications. This ability is especially significant during treatment of glioblastoma, where tumors can develop and extend into sensitive tissues such as venous sinuses, the brain stem, or deep cerebellar nuclei. These obstacles frequently prevent the ability to locally deliver drugs, and can even render resection impossible^1^.

Thus, the treatment of deeply embedded cancerous tumors in the brain via a compartmental therapy approach, specifically but not limited to Convection-Enhanced Delivery (CED), characterizes a specific clinical application where needle steering with very tight curvature would be highly effective. CED is a targeted drug delivery technique to treat various conditions in the brain. This technique uses a pressure gradient to deliver pharmaceuticals more successfully across the blood-brain barrier^2^. Another application where tight needle steering would provide clinical benefits is in radiofrequency ablation (RFA) of liver tumors. In RFA, a tumor or other target tissue is thermally destroyed by heat induced by high frequency alternating current, applied at the end effector of a small electrode^3^. This technique is often hindered by the maneuverability of the ablation needle; Adebar *et al*. specifically pointed out the need for tighter needle steering in order to target the highest number of points per entry wound as possible, reducing the risk of hemorrhaging^4^.

Although needle steering technology has not become widespread in the medical world, there exist numerous other medical procedures that would benefit from this technology. In order to effectively estimate the potential for needle steering technology, a study was conducted by Jong *et al*.^5^. This study was conducted at the Annual Meeting of Cardiovascular and Interventional Radiology Society of Europe in 2016, and surveyed 153 attendants. Although all respondents had at least 1 year of experience clinically using needles, the majority of respondents had over 10 years of experience using needles in clinical applications. Ultimately, it was found that 94% of respondents agree that needle steering would be helpful to correct unwanted needle bending; 84% of respondents find needle steering to be a useful tool for steering around anatomical obstacles; and only 2% of respondents do not believe that steerable needles would make new interventions possible^6–9^.

We propose a novel needle steering method that contrasts with both conventional needle steering and magnetically steered catheters and continuum manipulators by utilizing only magnetic forces and torques to both steer and advance a sharp magnetic tip. This method allows for arbitrarily soft shafts, as the needle is magnetically pulled rather than mechanically pushed. This eliminates the very possibility of buckling during insertion as well as restraints on minimal radius of curvature.

In traditional needle steering techniques, the curvature is achieved through the use of an asymmetrical beveled tip. Forces between tissue and the needle tip result in deflection of the needle. As the needle tip is pushed forward, it also moves slightly sideways, motivated by the radial component of the force acting on the tip. The magnitude of this sideways movement depends on the tip geometry, needle stiffness, tissue stiffness, bevel angle, and other properties of the needle-tissue interactions. The needle is rotated at the base to control the orientation of the tip, thus rotating the direction of the asymmetric force, providing controllability of the needle tip’s trajectory. This asymmetric tip may be beveled, complex, active, inactive, programmable, composite, or articulated^4,10,11^.

Advances in needle steering techniques are often focused on innovation of the mechanical needle design and its mode of manipulation. These techniques can be classified into two categories: passive and active^11^. Base manipulation^12^, rotating beveled needle^13^, pre-curved needle tip^14^, pre-bent needle tip^15,16^, notched shaft^17^, and other techniques employing passive needle modifications fall within the first category^11,18,19^. Khadem *et al*. used a notched needle tip to achieve a minimum radius of curvature of 171 mm^17^. Majewicz *et al*. achieved radius of curvature of 34 mm using a pre-bent needle tip^16^. Wedlick and Okamura used precurved needles to achieve a minimal radius of curvature of about 15.5 mm^14^, however the authors indicate that this method of needle fabrication is not perfectly repeatable.

Active needle steering techniques include telescoping cannula^20,21^, programmable bevel^22,23^, tissue manipulation^24^, and controlled articulating tip^4,25^. Van de Berg *et al*. used a needle having four actuation cables running alongside the shaft and connecting to the tip to alter the tip’s angle, and thus, achieved a radius of curvature of 181 mm^25^. Burrows *et al*. used a complex needle having four independently actuated interlocked shafts that could be extended or retracted to alter the tip geometry, achieving a radius of curvature down to 58 mm^23^. Adebar *et al*. placed an actuated hinge near the needle tip and were able to produce turning radii under 50 mm^4^.

According to the previously outlined categorization scheme, the proposed method of needle steering would be considered passive. Although it differs fundamentally from traditional needle steering techniques, it does not operate by moving parts and requires only control of the core system. Arguably, the device is not a catheter as it is intended only to penetrate tissue, but is a continuum manipulator.

Magnetic control of both soft and rigid bodies has been successfully conducted before^26–28^. Electromagnetic sensing and actuation have also been specifically used for needle steering. Cabreros *et al*. used magnetic actuation to vibrate small permanent magnets inside a needle shaft to produce vibrations for ultrasound needle tracking^29^. Dencker *et al*. performed electromagnetic needle tracking by including a magnetic sensor inside a needle tip^30^. Wang *et al*. steered a needle with a magnetic head by using magnetic fields to orient the head and applying mechanical force at the base of the needle for insertion^31^.

In addition, magnetically steered catheters and continuum manipulators have been extensively researched. Catheters are blunt, flexible rods generally meant to maneuver inside the existing channels in the body, to perform a host of tasks. Magnetically steered catheters and continuum manipulators use magnetic field generation systems to manipulate the device’s end effector, which houses a permanent magnet. These devices are inserted by hand^32^, or by using an automated advancer device^33–35^.

In its most general form, the device presented by Edellman *et al*. presents the essential magnetically steered catheter and continuum manipulator: the device utilizes one or more permanent magnets in its tip, manipulated with an 8 coil magnetic generation system, and utilizing an automated advancer at the base^35^. Chautems *et al*. presented a magnetically re-formable catheter, composed of a magnetic tip followed by a segment containing a low melting point alloy; the alloy could be melted in certain locations along the shaft and magnetically deformed via manipulation of the permanent magnet in the tip, then the alloy solidified and the new configuration thus held^36^. Contemporary research in magnetically steered catheters is quickly advancing towards applications in the brain^37,38^, lung^34^, eye^39^, and heart^40^. The insertion mechanism in all of these devices is however still reliant on axial forces along the shaft: actuated by hand-pushing or by an automated advancer pushing at the base. A taxonomy of the devices mentioned are included in Table S1.

In fact, all prior methods of both needle steering and magnetically steered catheters and continuum manipulators rely on the use of mechanical insertion, or “pushing” to move the device further into the tissue. The main contribution of this research is the shift from “pushing” the inserted robot, to “pulling” it; utilizing only magnetic torques and forces at the tip to both steer and advance the robot. This study highlights how the proposed technique addresses the limitations of prior needle steering, and compares it to prior magnetic steering of catheters and continuum manipulators. In particular, the limitations of past needle steering techniques that are discussed include torsion effects on the shaft, rotational tissue damage, and needle deflection at tissue boundaries. In addition, buckling in the shaft and limited radius of curvature^9,18,41,42^ that are common between the traditional needle steering method, magnetically steered actuators, and continuum manipulators are also discussed. Notably, each limitation is described followed by a discussion of how the proposed method eliminates it. The issues related to the needle deflection at tissue boundaries and limited radius of curvature which require further experimental investigation are described in the results section. On the other hand, buckling and torsion effects as well as excess tissue damage that are inherently eliminated by the proposed method are explained in the discussion section. Finally, safety and scalability of the system as they relate to clinical applications are also deliberated in the discussion section.

## Results

The proposed method replaces the conventional load-bearing shaft needle with an elastic shaft attached to a magnetic needle tip and utilizes electromagnetic pulling actuation instead of mechanical pushing actuation. This change in needle shaft design and actuation method leads to the elimination of all of the above-mentioned issues with conventional needle steering, magnetically steered catheters, and continuum manipulators. Here, we specifically discuss how the needle deflection at tissue boundaries and limited radius curvature are addressed by the proposed method. Next, we present experiments designed to demonstrate the ability of this method to follow complex trajectories.

### Tissue boundary deflection

In many medical needle applications, the ability to maintain a straight path can be integral to the success of the procedure^43^. However, in conventional needle steering methods, needle tips can experience significant deflection while crossing a membrane during insertion at shallow angles of attack. Reed *et al*. reported a tip deviation of over 1 cm between the final tip position of an insertion through a simulated membrane and a control insertion^8^. In the interface experiments presented in this paper, however, the needle is able to correct its trajectory and follow the desired path even when puncturing a tissue interface.

**Figure 1:**
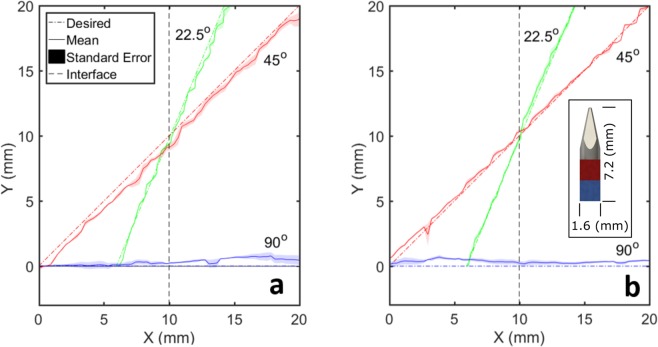
Actual paths followed by needle tip over all experimental cases in (**a)** stiff to soft tissue transitions and (**b)** soft to stiff tissue transitions.

Table 1 is a summary of the experimental cases, the associated deviation of the experimental mean from the ideal path as shown in Fig. 1, and root mean square (RMS) of the errors in all trials for each case. The ideal path is defined as a line following the defined angle of attack from the needle’s starting position. Transition type describes the starting and ending tissue types for the experiment. Desired angle of attack (AoA) is the angle along which forces are applied to motivate the magnetic needle tip to move.

**Table 1:** Maximum deviation of experimental mean from defined path and RMS of the error for interface experiments. The overall RMS error of these experiments is 1.2 mm.

Case	Transition Type	Desired AoA	Max. Deviation of the Mean from the Path	RMS of the error
1	Soft to Stiff	90 Degrees	0.8 mm	0.6 mm
2	Soft to Stiff	45 Degrees	2.3 mm	0.8 mm
3	Soft to Stiff	22.5 Degrees	0.5 mm	0.7 mm
4	Stiff to Soft	90 Degrees	0.8 mm	0.5 mm
5	Stiff to Soft	45 Degrees	0.8 mm	1.4 mm
6	Stiff to Soft	22.5 Degrees	1.0 mm	2.1 mm

The needle tip chosen for use in experiments had a diameter of 1.59 mm. All deviations of experimental means from the desired paths are less than 2.3 mm with the overall RMS error of 1.2 mm, demonstrating the ability of our proposed method to greatly improve upon the issue of tissue boundary deflection. In addition, these interface experiments showcase the ability of the proposed method to maintain a straight path through a uniform medium as well as an interface of tissues with different stiffness.

### Restricted radius of curvature

The radius of curvature in conventional needle steering methods is constant and dependent on the characteristics of the particular needle and tissue pair interactions. The result is that for any given tissue/needle combination, only one radius of curvature is attainable without the use of complex mechanisms such as manipulating the tissue via external forces or duty cycling. Conventional needle steering methods utilize the interaction forces between tissue and the needle tip in order to achieve steering. Conversely, this proposed method uses magnetically generated forces and torques to achieve certain radii of curvature. In addition, the lack of compressive forces on the needle shaft allows it to be made arbitrarily soft and flexible, theoretically eliminating restraints on minimal radius of curvature experienced in magnetically steered catheters and continuum manipulators. The ability to achieve a small radius of curvature is important because larger radius of curvatures restrict the reachable workspace.

In order to demonstrate the performance of the proposed needle steering technique in making “sharp” turns without duty cycling, three different radii of curvature have been tested: 10.2 mm, 20.3 mm, and 30.5 mm in both soft and stiff phantom tissues. Table 2 presents the maximum deviation of the experimental mean from the desired path and RMS of the errors in all trials for each radius of curvature and phantom tissue material. The needle performs better in the soft phantom tissue than stiff, achieving maximum deviations of 0.8 mm and RMS error of 1.3 mm or less. Overall, for the chosen experimental needle tip having a diameter of 1.59 mm, the predefined curved paths are achieved with maximum deviations under 1.5 mm. The RMS error for all of the radius of curvature experiments is also 1.4 mm.

**Table 2:** Maximum deviation of experimental mean from defined path and RMS of the error for radius of curvature experiments. The overall RMS error of these experiments is 1.4 mm.

Phantom Tissue Material	Radius of Curvature	Max. Deviation of the Mean from the Path	RMS of the error
Soft	10.2 mm	0.8 mm	1.3 mm
Soft	20.3 mm	0.7 mm	1.3 mm
Soft	30.5 mm	0.6 mm	1.2 mm
Stiff	10.2 mm	0.8 mm	1.4 mm
Stiff	20.3 mm	1.2 mm	1.5 mm
Stiff	30.5 mm	1.5 mm	1.7 mm

**Figure 2:**
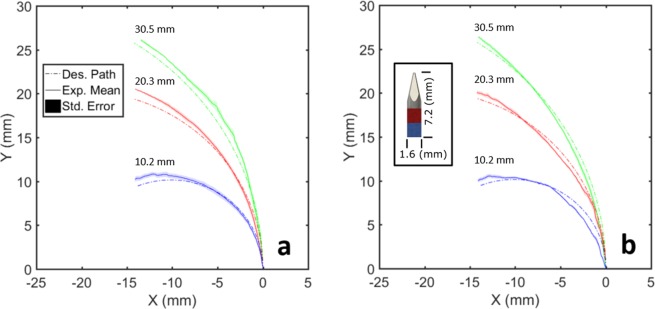
The position (*x*, *y*) of the magnetic needle tip as it follows the defined circular path in (**a)** stiff tissue and (**b)** soft tissue.

The minimal repeatable, reliable radius of curvature achieved by the proposed magnetic needle steering method is 10.2 mm as shown in Fig. 2. This limit is due to the radius of curvature approaching the total length of the needle. As the needle tip length (7.2 mm) and radius of curvature converge, the needle tends to “cut” the tissue with the shaft during rotation around the curve, instead of piercing the tissue during forward motion. Such behavior causes excessive damage to the tissue, and is thus not desirable for needle steering. Smaller radii of curvature would be possible with a shorter needle, but a similar limit will be reached as the curvature approaches the needle tip length.

The minimum repeatable radius of curvature achieved by conventional needle steering methods has been reported to be around 34 mm^16^. However, due to the impact of the medium on radius of curvature, a direct comparison between this curvature and the proposed method’s minimum radius of curvature is unreliable. In order to provide such a direct comparison, we conducted an experiment utilizing this conventional pre-bent, beveled needle design moving through the same mediums utilized in this study. Since the stiffness of the gels are too low to apply forces large enough to curve the needle, it tends to move on a straight path, compared to a minimum 10.2 mm radius of curvature achieved in both gels using our system.

### Complex trajectory

Although Table 2 shows the ability of this system to take variable radii of curvature in a 2D plane, it is necessary to be able to maneuver in all three dimensions (3D) for clinical applications. This system is capable of generating a magnetic field in any direction which makes it possible for the needle to travel on any path. This also enables any radius of curvature between the minimum possible radius and the straight line to be taken in 3D, as demonstrated by the experiments in this section.

**Figure 3:**
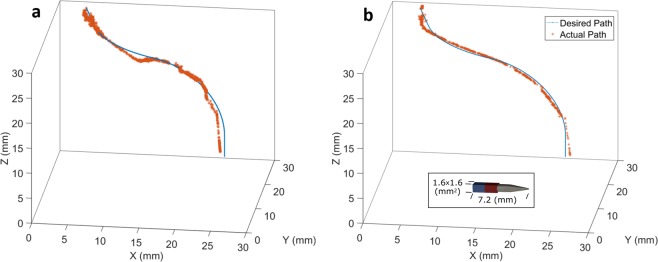
The position (*x*, *y, z*) of the magnetic needle tip as it follows the defined complex path in (**a)** stiff tissue and (**b)** soft tissue.

The results of these complex 3D path experiments are shown in Fig. 3. The 1.59 mm diameter needle is able to accurately follow the predefined path with a maximum error of 2.4 mm in the soft gel, and 2.7 mm in the stiff gel. The RMS error in the soft and stiff gels is also 0.9 mm and 0.4 mm, respectively. As shown by these experiments, this proposed method allows for steering the needle through mediums on complex 3D paths without causing any of the excess tissue damage that would be caused by conventional needle steering methods.

## Discussion

By presenting a new method of needle steering, several drawbacks of the conventional needle steering techniques as well as magnetically actuated catheters and continuum manipulators have been eliminated and various improvements to the state of the art have been demonstrated. Compression effects such as buckling of the needle shaft and torsional effects in the shaft are significant drawbacks that cause unnecessary damage in tissue, inaccuracies in control, and increased model complexity. These drawbacks are done away with in the proposed magnetic needle steering method by design, as the elastic shaft is not load bearing in compression or torsion. In addition, the proposed method does not suffer from significant deflections when crossing a tissue boundary, and can reliably move along a predefined straight path through two different tissues and the interface boundary between them. Moreover, repeatable path following at various radii of curvature down to 10.2 mm is shown to be possible in different tissue materials without requiring the use of duty cycling. It is also shown that the proposed method is capable of steering the needle on complex paths with different radii of curvature in 3D, without duty cycling. Here, we discuss buckling and torsion effects in addition to excess tissue damage that are inherently eliminated by the proposed approach. Next, we address issues related to safety and scalability of the system as they relate to healthcare applications.

### Buckling

Buckling is one of the inherent issues with conventional needle steering as well as magnetically steered catheters and continuum manipulators. In conventional needle steering methods, flexible needles need to be supported at the base to prevent buckling in the needle shaft at the entry point as it penetrates the tissue^9^. Too much force exerted on the needle shaft during insertion can cause the needle shaft to slice through the tissue laterally, even without buckling^8^. Narayan *et al*. used a model to detect buckling events that occur when a needle tip encounters an object it cannot penetrate, demonstrating that buckling can occur due to collisions and inhomogeneity in the tissue^44^. Buckling is a concern in all of the aforementioned methods because the shaft is actuated by a compression force acting through it. Indeed, the fact that the shaft must be able to withstand significant compression stress conflicts with the necessity that the shaft also must be flexible enough to permit steering. To address this issue in the proposed method, the needle shaft is replaced by an elastic shaft that is not load bearing. By pulling the needle tip using magnetic forces instead of pushing at the base of a load bearing shaft, buckling is no longer a concern, and the conflict between shaft stiffness and flexibility is eliminated. Additionally, eliminating this conflict has the potential to reduce restrictions on achievable radius of curvature caused by load bearing shafts.

### Torsion in the needle shaft

Complex torsional effects along the shaft is another inherent drawback of conventional needle steering methods. This effect is caused by friction between the needle shaft and the tissue, and can cause the base and tip to rotate out of sync.

The discrepancy between the base and tip rotations can be as high as 45 degrees for a needle insertion of only 10 cm which leads to poor performance^41,42^. Although there have been approaches to improve the performance of the conventional needle steering methods through modelling and controlled compensation of these torsional effects, this comes at the expense of additional complexity and difficulty in modelling and control^45,46^. The proposed magnetically actuated method does not rely on the asymmetry of the needle tip for steering and thus, does not require control over rotation about the needle’s axis, eliminating the difficulties caused by these torsional effects.

### Rotational tissue damage

To obtain various radii of curvature as well as straight path in traditional needle steering, the needle’s rotation must follow a controlled and repeating pattern^47^. This method is called duty cycling. For pre-bent, pre-curved, and some complex needle designs, duty-cycled rotation of the needle tip can cause significant additional tissue damage during insertion due to the asymmetric tip^18^. The proposed magnetic steering method does not require duty cycling to achieve varying radii of curvature, and instead applies magnetic torques and forces in the specified direction of the magnetic field and gradient, respectively. Therefore, this method does not require rotation about the needle’s axis to achieve variable radii of curvature, precluding the tissue damage induced by duty-cycled tip rotation. Magnetically guided catheters and continuum manipulators also operate this way, eliminating excess damage to the tissue from duty cycling.

### Safety and scalability of the system

There are a number of safety concerns that could arise when using magnetically actuated surgical devices. In the case where this system is scaled up and emits much higher magnetic gradients, there are concerns regarding prolonged human exposure to powerful magnetic fields. However, prolonged exposure to 8 Tesla magnetic fields has been thoroughly studied and there has been no significant impact on vital signs^48^. In addition, there may be concerns about uncontrolled needle movement within the body which could cause unnecessary tissue damage. Two mechanisms of safety can be employed to combat this concern during clinical use. One is removing the external magnetic gradient which can be done by simply shutting off the power to the electromagnets. A second mechanism is using a motorized spool that can slowly release the tether/tube at a constant rate to prevent the needle from jerking forward.

The scalability of this system for clinical use is another issue that must be addressed due to size and force constraints of the proposed electromagnetic system. The possibility of adding the missing degree of freedom (rotation about the magnet’s main axis) could be investigated in order to reduce required needle insertion forces^49,50^. In addition, utilizing a system of permanent magnets could prove to be a promising alternative for steering the needle. Larger magnetic forces can be achieved using permanent magnets while taking up much smaller size compared to similar strength electromagnets. There are several studies on using permanent magnets to control the position and orientation of magnetized bodies which can be adapted for steering magnetized needles inside tissues^51–53^.

In the case of using permanent magnets to generate the actuating magnetic fields and gradients, achieving the safety of a reliable shut-off switch is a vital concern. The authors envision such a system utilizing a deployable magnetic shielding, to instantly remove magnetic field and gradient acting on the needle tip. Mahoney *et al*. describe attaching a permanent magnet to a robotic arm^52^. Such a system is diagrammed in Fig. S5, which illustrates this potentially clinically viable setup. It is notable that such a system would have a greatly increased workspace^54^.

For theoretical clinical procedures based on the proposed method and utilizing a more powerful magnetic field generation system to steer a needle tip through tissue, position feedback must be obtained non-visually. Ultrasound imaging has been used for feedback in traditional needle steering^55^. Ultrasound does not interfere with magnetic fields, and as such, it would be a valid option for position feedback in a clinical system. An extension of ultrasound guidance is a technique called Image Fusion, in which a previously recorded CT or MRI scan is referenced alongside the real-time ultrasound feedback. This technique has been proven viable for clinical needle steering based biopsy in two cases, as presented by Dencker *et al*.^30^. Ultimately, the proposed magnetic needle steering method is promising for its potential ease of development for clinical applications, as the technology for human-scale medical magnets is well established. However, the focus of this study is more on introducing a new needle steering technique and providing proof of concept. This technology would certainly need to be further developed to make it readily feasible for clinical use.

## Methods

### Magnetic coil system

An electromagnetic field generation system (Fig. S1) consisting of an array of eight stationary, current controlled coils is used to produce and manipulate the actuating magnetic field and magnetic gradient^28^. This system enables five degrees of freedom (DOF) (3-DOF of translation and 2-DOF of rotation; the rotation about the magnet’s main axis is not achievable with this system) magnetic control of a small magnetic body in a 33 × 33 × 33 mm^3^ workspace. It is important to note this particular geometric layout of the coils is not needed to achieve five degrees of freedom, as established by Pourkand *et al*.^56^. The geometric design is however chosen for simplicity of construction and implementation. Other systems have been designed for manipulation of magnetic robots such as BigMag^57,58^, and Octomag^28^ upon which the current system was based. The proposed method is not restricted to a system arranged in any particular way, and can be achieved by any magnetic field generation system that provides sufficient degrees of freedom for the desired task.

Each coil is composed of a solid iron core and 712 wraps of 14 gauge copper wire in 6 layers. Two power supplies are used: a TITAN F1208 capable of providing 60 A and up to 1200 W of electrical power, and an eFuel PSU50A V2 capable of providing 50 A and up to 1200 W of electrical power. Four Sabertooth 2 × 25 dual motor drivers are used as amplifiers between the power supplies and coils. Amplifiers are connected to a Sensoray Multifunction analog/digital I/O-Model 826 data acquisition card (DAQ) to receive the control inputs.

In sufficiently soft mediums, a magnetized body becomes aligned with magnetic flux density, as the environmental resistance to rotation is small. In such cases, magnetic flux density can be used to define the desired orientation of the needle. Force applied on the needle is determined by the magnetic gradient in each direction and the magnetization of the needle tip. Both magnetic flux density and gradient are produced by the linear combination of each electromagnetic coil’s contribution to the net magnetic field. These contributions were determined through a calibration process that characterized each coil’s magnetic field contribution in $x$, $y$, and $z$ at 1A. The flux and force can be defined and the current through each coil (${i}_{0}\,\mathrm{..}.\,{i}_{7}$) calculated as,

1 $$[\begin{array}{l}{i}_{0}\\ \vdots \\ {i}_{7}\end{array}]={[\begin{array}{l}\beta ({\bf{P}})\\ {{\bf{M}}}^{T}{\beta }_{x}({\bf{P}})\\ {{\bf{M}}}^{T}{\beta }_{y}({\bf{P}})\\ {{\bf{M}}}^{T}{\beta }_{z}({\bf{P}})\end{array}]}^{\dagger }\,[\begin{array}{l}{{\bf{B}}}_{desired}\\ {{\bf{F}}}_{desired}\end{array}]={A}_{B,F}{({\bf{M}},{\bf{P}})}^{\dagger }\,[\begin{array}{l}{{\bf{B}}}_{desired}\\ {{\bf{F}}}_{desired}\end{array}],$$

where *β* represents the magnetic field contribution of each of the eight electromagnetic coils at a particular position **P**. ${\beta }_{x},{\beta }_{y},{\beta }_{z}$ are the gradients of the calibration matrix in each direction at the same position **P**, ${{\bf{B}}}_{desired}$ is the desired flux density in each direction, ${{\bf{F}}}_{desired}$ is the desired force in each direction, **M** is the magnetization vector of the needle tip, and ^†^ represents the pseudo-inverse. In the case of the magnetic coil system used for these experiments, the maximum magnetic flux density that can be produced is 32 mT, and the maximum magnetic gradient is 1.4 Tm^−1^. These limitations exist to avoid overheating the electromagnetic coils: each coil’s current is limited to 12A.

### Phantom tissue selection and fabrication

Two phantom tissue materials are selected carefully, considering the strength of the magnetic coil system and the requirement of transparency for the object tracking software. Due to limitations on magnetic field strength, the chosen phantoms have lower stiffness than that of some human tissues (1.96 kPa for the soft gel, and 2.70 kPa for the stiff gel). However, based on research done by Murphy *et al*. on the stiffness of brain tissue between patients with Alzhiemers disease and a control group, the stiffness of various brain structures ranged from 2.42 to 2.82 kPa, which is very close to the tissues studied in this research^59^. Furthermore, in a study by Koch *et al*., the stiffness of healthy liver tissue was found to be 5 kPa, and found to be 10kPa for patients with obesity, type 2 diabetes, and those undergoing intensive care^60^. The experiments show that a sharp magnetic tip attached to an arbitrarily flexible shaft can be magnetically pulled and steered through tissue along a defined path, without the need for an advancer, insertion device, push-wire, or other device pushing or otherwise anchoring the shaft at its base. The research is justified noting that by scaling the system to produce sufficiently powerful magnetic fields and gradients, the technique demonstrated in this proof of concept could be used for steering a sharp magnetic tip through any tissue in the body. In this study, the two phantom tissues are required to simulate the needle passing through one tissue into another, and to test the ability of the system to achieve a consistent radius of curvature for the needle in different materials. The two tissue materials chosen are referred to as “soft” and “stiff” throughout the paper.

The soft phantom tissue is a mixture of 1.2 g of agar gelling powder and 0.08 g of pure agarose powder per 100 mL of distilled water. The stiff phantom tissue is comprised of 1.6 g of agar gelling powder and 35 *μ*g of pure charcoal per 100 mL of distilled water. The soft tissue is characterized by a much greater capacity for elastic deformation before rupture and stiffness of 1.96 kPa; contrasting with the stiff tissue, which ruptures before significant deformations are observed and has a stiffness of 2.70 kPa. This mechanical difference can be seen in the results obtained in the experiments. The agar gelling powder is produced by Nitta-Gelatin, and is composed of 11% locust bean gum, 9% carrageenan, 3% phosphorus acid, and 77% glucose. The agarose powder is molecular biology grade Agarose LE powder from Benchmark Scientific. The pure charcoal powder is produced by General’s. Weights are measured using a US Solid Digital Analytical Balance USS-DB55. Both soft and stiff phantom tissues are used in the needle characterization, tissue-tissue interface, radius of curvature, and 3D complex path experiments. These materials are chosen for their relatively quick set time and transparency. The concentrations of the soft and stiff phantom tissues are determined through several iterations of experiments during preliminary exercises exploring the coil system’s capabilities.

### Control scheme

The control of the system is outlined in Fig. S2. All experiments are performed in closed-loop, with visual feedback enabling the calculation of position errors for use in a multiple-input, multiple-output system of proportional-integral-derivative (PID) controllers. Two Logitech c920 cameras mounted on the top and side of the system are used to detect three dimensional position information of the colored tip through image processing using the OpenCV library for C++. A system of PID controllers manipulate magnetic gradient and thus force, to control $x$, $y$, and $z$ position. The magnetic field is applied based on desired needle orientation. The output force and magnetic field is multiplied by a matrix ${{A}_{B,F}}^{\dagger }$ to produce the currents to send to the coils of the system. The matrix ${{A}_{B,F}}^{\dagger }$ contains the terms *β* and **M**. To calculate *β* a vector representation of the coils’ unit contribution to the magnetic field and gradient, the position **P** is used to reference a calibration matrix. The magnetization vector of the magnetic tip **M**, is assumed to be in the same direction as the magnetic field **B**. In each iteration of the control loop, it is the multiplication of ${{A}_{B,F}}^{\dagger }$ and the output force and magnetic field that results in the current that is sent to the coils.

In the radius of curvature and complex 3D path experiments, the paths are first described via one function or a series of functions, respectively, that are completely contained within the workspace domain. The path curves are broken down into segments based on a specified arc length, and the segment end-point coordinates are stored in an array. This arc length is specified to be 0.03 mm in the present research, and can be made arbitrarily small, limited only by computational capabilities. During execution, magnetic force is applied to move the needle tip towards the points in the array sequentially, and magnetic torque applied to rotate the tip to face the direction of the next point in the array. In the event that the tip deviates from the path or moves beyond the next point in the sequence, the closest point on the path is found and treated as the set point. In this way, not every point in the sequence must be reached, and no back-tracking occurs. This path following process is illustrated in Fig. S3. The magnetic field direction is always set to be in the direction of the next point in the path. The facing of the needle is then assumed to be in the direction of the magnetic field, as previously described.

### Needle selection and fabrication

It is necessary to develop a needle that embodies two key characteristics that affect the amount of force required to move it through the phantom tissues: first, the needle must be sufficiently magnetized to allow for enough force to be generated by the coil system; second, the needle must have a sharp tip to further reduce required forces. A very small permanent magnet acts as the magnetic element, and a needle tip adhered with a cyanoacrylate adhesive to the magnet acts as the sharp element. The magnet is painted red and blue with a dibutyl phthalate/toluene/formaldehyde resin paint so that the tip and rear of the needle can be detected and differentiated via a color detection software. The colorant and adhesive are chosen to be inert with the phantom tissue material.

The chosen magnet has dimensions of 3.2 mm by 1.6 mm by 1.6 mm. The magnet is grade N42, nickel coated, and weighs 0.060 g. It has a residual flux density of 1.32 Teslas. From the dimensions and residual flux density, it is known that the magnet has a surface field of 0.6353 Teslas. This rectangular magnet is chosen against cylindrical magnets because of availability at the size and grade.

**Figure 4:**
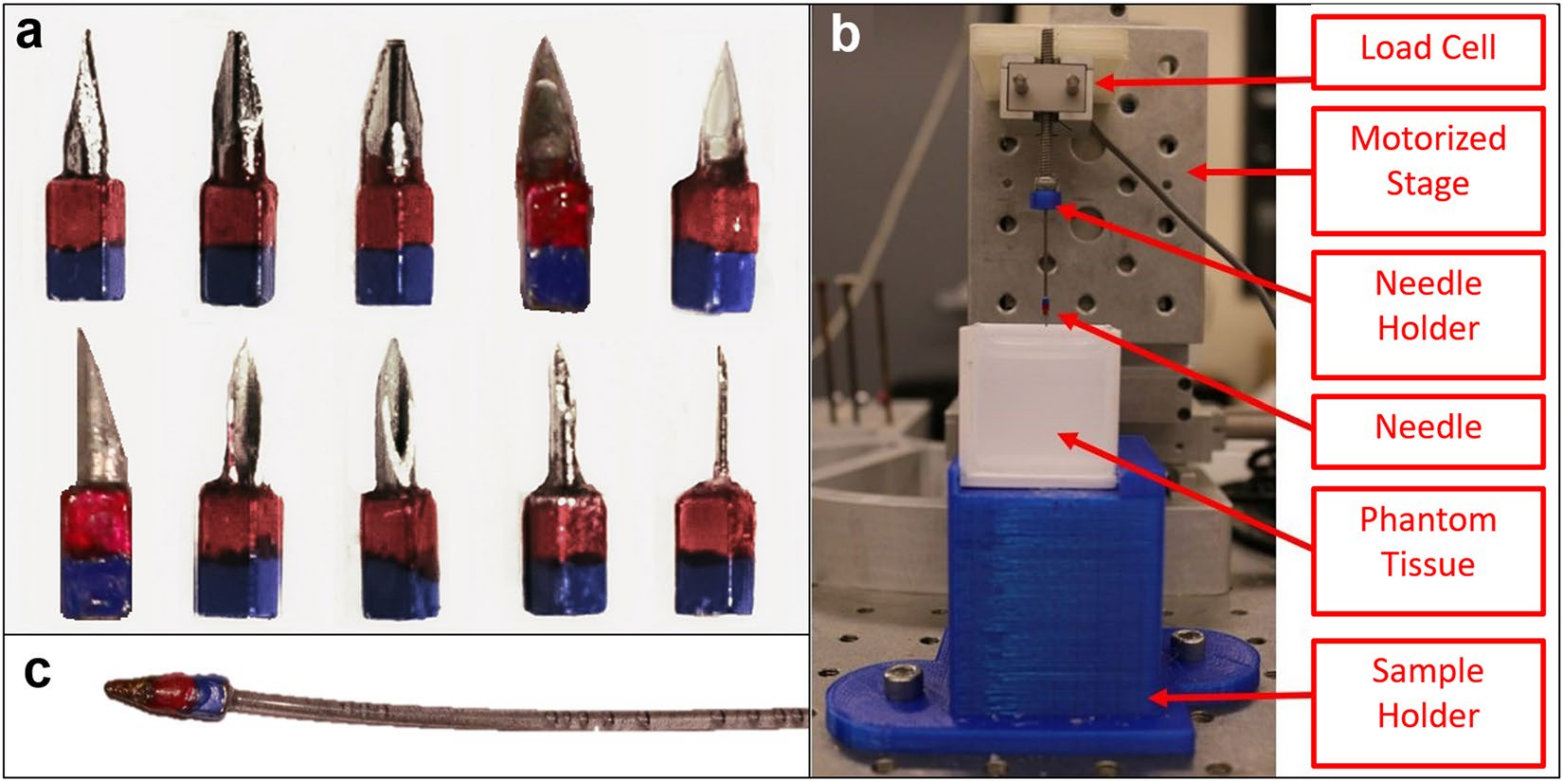
(**a)** From left: (top row) 0.88 mm diamond shaped tip (88 N), 1.2 mm diamond shaped tip (12 N), 1.6 mm diamond shaped tip (16 N), 14 gauge medical needle (14 G), 16 gauge medical needle (16 G), (bottom row) 18 gauge medical needle (18 G), 19 gauge medical needle (19 G), 20 gauge medical needle (20 G), 25 gauge medical needle (25 G), 30 gauge medical needle (30 G). (**b)** Experimental setup for the needle characterization experiments. (**c)** Needle with flexible shaft.

Ten needle tips are selected and fabricated for characterization. Seven tips are selected to be cut from various medical needle tips, and three tips are selected from standardized diamond-shaped needles. These fabricated needle tips are displayed in Fig. 4a. The needle tips are cut using a diamond dusted cutting disc on a hand-held dremel and a 3D printed holder, to be 4 mm with a tolerance of ±10%. Each tip is attached to the magnet, composing a full needle, 7.2 mm in length.

To evaluate different needle tips when puncturing and moving through the phantom tissues, the force profiles of each needle tip in each phantom tissue is compared. This characterization experiment is done in a force measurement setup consisting of a Thorlabs MT1-Z825V actuated 3-axis stage, a Transducer Techniques GSO-50 load cell, a Newport Integrity 3 vibration control table, a Thorlabs TDC001 motor controller, and an Interface USB enabled DAQ. Via custom assemblies, the needle is mounted to the force sensor, which is mounted to the translational stage. This setup is illustrated in Fig. 4b. The stage is programmed to move at a constant velocity of 0.5 mm per second such that the needle punctures the phantom tissue and moves through the tissue for some additional distance, totalling a translation of 25 mm. Force is measured throughout the entire process and categorized in three subsets: needle tip puncture and insertion, magnet insertion, and interior movement. These three regions of interest in the needle insertion force profiles are characterized individually for each needle/tissue pair in Fig. S4 in the supplementary materials. These regions correspond to two peaks that occur during the puncturing process, and the steady state force that is observed when moving through the tissue. The first peak coincides with the initial puncture of the needle tip into the phantom tissue surface, labeled “needle tip insertion”. Sharper needles experience smaller force values in this region. The second peak observed coincides with the insertion of the magnet body into the phantom tissue. Needle tips with a smaller diameter have a larger exposed magnet face and thus, experience greater force during this second peak, the “magnet insertion”. The third region is the internal movement force; the force required to move through the tissue while the entire body is inside, labeled the “steady state internal movement” region.

Figure S4 compares the performance of each needle. The maximum force for each needle observed at each of the three aforementioned regions is shown. The initial puncture force ranges from about 2 mN to about 18 mN in the soft phantom tissue, and from about 1 mN to 6 mN in the stiff tissue. The force required for the insertion of the magnet ranges from 3 mN to about 12 mN in the soft tissue, and from 2 mN to about 18 mN in the stiff tissue. The forces required for internal movement through the phantom tissues ranges from 0.6 mN to 1.3 mN for the soft tissue and 1.4 mN to 2.6 mN for the stiff tissue.

The fact that the 1.6 mm diamond-point tipped needle had relatively small magnet insertion force in both tissues makes sense, as there is the least amount of exposed magnet surface for this needle design. Interestingly, this tip also proved to have the best internal movement force profile over all experiments, requiring the least internal movement force in the soft tissue and the second least internal movement force in the stiff tissue. As the experiments and intended use of this technology take place inside tissues (where the entire needle is inside the tissue), this needle is chosen as the optimal among the tips tested.

These designs for the magnetic needles are not optimized for clinical performance in that the large magnet shoulder will cause damage and an increase in required penetration forces. They are simply designed to demonstrate the novel magnetic steering and actuation technique. More research is certainly needed on magnetic needle tip design to come to a configuration achieving minimal force profiles for future healthcare applications.

The flexible needle shaft is not fixed at the base, there is no advancer device to control insertion, there is no push-wire, and the shaft is not advanced by hand nor by motor. Instead, the needle is actuated, advanced, and steered solely by magnetic forces and torques. The tail of the flexible shaft simply protrudes from the entry hole in the workspace and is laid out on a water-lubricated surface to reduce friction effects that may restrict the needle’s maneuverability. As the magnetic forces pull the needle tip further into the tissue, more of the flexible shaft is pulled into the workspace as needed. The forces involved in dragging the shaft on the lubricated surface did not halt the ability of the needle to maneuver in the workspace.

### Tissue-tissue interface experiment

In these experiments, a magnetic needle tip is forced to pass through the interface plane of two different phantom tissues at three different angles of attack. The needle is steered via a closed loop controller receiving visual feedback from two cameras and controlling the current through each coil. The needle’s position is measured over the course of the experiment. Six experimental cases are designed: three angles of attack (90 degrees, 45 degrees, and 22.5 degrees), for two transition types (soft-to-stiff and stiff-to-soft tissue transitions). The coil system generates a magnetic field which aligns the needle to the specified angle for the particular experimental case and simultaneously generates a pulsing magnetic field gradient to apply force to the needle in the same direction. A PID controller is employed to manipulate the magnitude of the force pulses. The program is stopped manually after the needle has sufficiently crossed the gel interface.

### Radius of curvature experiments

These experiments demonstrate the system’s ability to achieve variable radii of curvature without excessively damaging the tissue. In addition, the minimum achievable radius of curvature in both soft and stiff phantom gels are also measured through these experiments. The experiment consists of the needle tip following three different predefined circular paths (i.e., 10.2 mm, 20.3 mm, and 30.5 mm radii of curvature) through the two different phantom tissue materials, under closed-loop position feedback control. A circular path in a 2D plane with respect to the needle tip’s current position is generated. The system then uses visual feedback in conjunction with two PID controllers; relating the error between the current position and the position of the next path point in $x$ and $y$ directions to the desired force in the respective direction. The system sequentially aligns the needle tip’s position to each point along the generated path. The needle’s orientation is assumed to be in-line with the applied magnetic field, which is an accurate assumption given a sufficiently powerful magnetic field.

### 3D complex path experiment

The proposed magnetic actuation method has 5-DOF capabilities and is not limited to planar path control. The path used to demonstrate 3D maneuverability without the excessive tissue damage caused by traditional methods starts with a straight section in $xy$ plane followed by a curved section with 20 mm radius of curvature. Next, the path starts to leave $xy$ plane by going on a curved section with radius of curvature of 12 mm. Finally, the path goes along the *z* axis on a straight line. Like the radius of curvature experiments, these experiments utilize visual feedback in conjunction with three PID controllers in $x$, $y$, and *z* directions. For increased visualization in this experiment, the shaft was dyed.

## Supplementary information

Supplementary Materials.

Supplementary Video.

## Data Availability

The authors declare that all data will be made available; either upon individual request or through a *Scientific Reports* preferred online repository, if needed.
