# Supplementary material for: Magnetic Needle Steering in Soft Phantom Tissue
Source: Sci Rep. 2020 Feb 12;10:2500. doi: 10.1038/s41598-020-59275-x (PMC7016187; doi:10.1038/s41598-020-59275-x)
Supplement: Supplementary file 1 — Supplementary Materials. [file 41598_2020_59275_MOESM1_ESM.pdf]

Magnetic Needle Steering in Soft Phantom Tissue

Supplementary Materials

Mahdi Ilami[1,+], Reza James Ahmed[1,+], Alex Petras[1], Borhan Beigzadeh[1,2], and HamidMarvi[1,*]
[1] Arizona State University, School for Engineering of Matter Transport and Energy (SEMTE), Tempe, 85287, USA
[2] Iran University of Science & Technology, School of Mechanical Engineering, Tehran, Iran
[*] corresponding author: hmarvi@asu.edu
[+] these authors contributed equally to this work

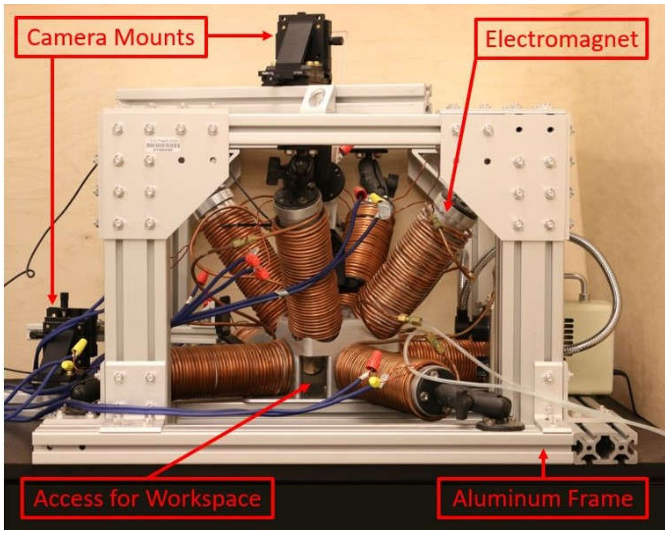

Figure S1. Electromagnetic coil system used to actuate magnetic needle tip. Two cameras are used to track the colored tip. The workspace cube is inserted through an access opening and placed at the center of the coil array.

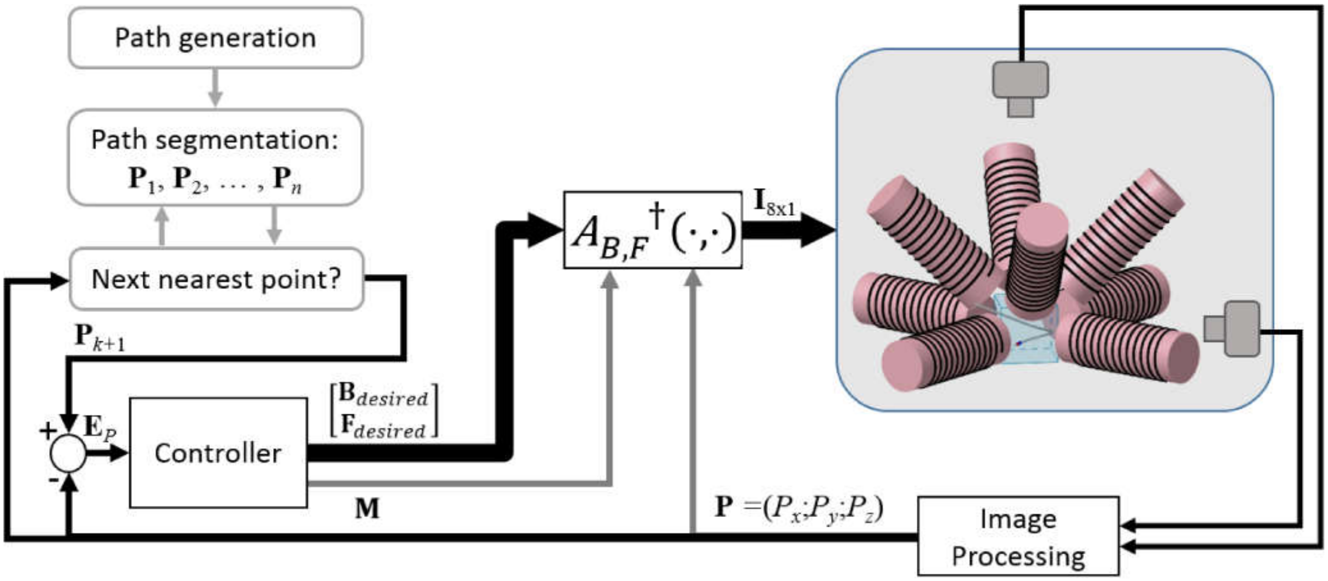

Figure S2. Block diagram outlining the control scheme of the magnetic field generation system to control the needle tip.

Table S1. Taxonomy on needle steering technologies

heightResearch Topic	Steering Method	Open<br>Closed Loop	Advancement System	Accuracy<br>(Presenting method / Value)
Robotic-Assisted Needle Steering Around Anatomical Obstacles Using Notched Steerable Needles[17]	Non-Magnetic	Closed	Human	Mean targeting accuracy / 1.2 mm
A Flexure-Based Steerable Needle: High Curvature With Reduced Tissue Damage[61]	Non-Magnetic	Open	Human	N/A
Evaluation of Robotic Needle Steering in ex vivo tissue[16]	Non-Magnetic	Closed	Mechanical	N/A
Characterization of pre-curved needles for steering in tissue[14]	Non-Magnetic	Open	Mechanical	N/A
Design of an actively controlled steerable needle with tendon actuation ad FBG-based shape sensing[25]	Non-Magnetic	Closed	Mechanical	Targeting accuracy 6.2±1.2 mm<br>Steering precision / 2.61.1 mm
Experimental characterization of a biologically inspired 3D steering needle[23]	Non-Magnetic	Closed	Mechanical	RMS path error / 0.9 mm
Methods for Improving the Curvature of steerable needles in biological tissue[4]	Non-Magnetic	Closed	Mechanical	Standard Deviation<br>radius of curvature / 4.9 mm
Feasibility Study of Robotic Needles with a Rotational Tip-Joint and Notch Patterns[62]	Non-magnetic	Closed	Mechanical	N/A
Improving guidewire-mediated steerability of a magnetically actuated flexible microrobot[40]	Magnetic	Open	Human	Maximum<br>error of tip alignment [a] / 6.2 degree
Magnetic Needle Guidance for Neurosurgery: Initial Design and Proof of Concept[37]	Magnetic	Open	Human	N/A
Magnetic Control of continuum devices[35]	Magnetic	Closed	Mechanical	RMS distance error / 0.42 mm
Magnetic Continuum Device with Variable Stiffness for Minimally Invasive Surgery[36]	Magnetic	Closed	Mechanical	N/A
Vision-based 3-D control of magnetically actuated catheter using BigMag-An array of mobile electromagnetic coils[57]	Magnetic	Closed	Mechanical	RMS trajectory error / 0.77 mm
A Magnetically Controlled soft microrobot steering a guidewire in a three-dimensional phantom vascular network[33]	Magnetic	Open	Mechanical	Percentage error between experiment and simulation / 11.5%
Magnetic Needle Steering in Soft Phantom Tissue	Magnetic	Closed	Magnetic	RMS error of interface Exp. / 1.4 mm<br>RMS error of radius of curvature Exp. / 1.2 mm

[a]The error in degrees between the actual alignment of the tip and the magnetic field

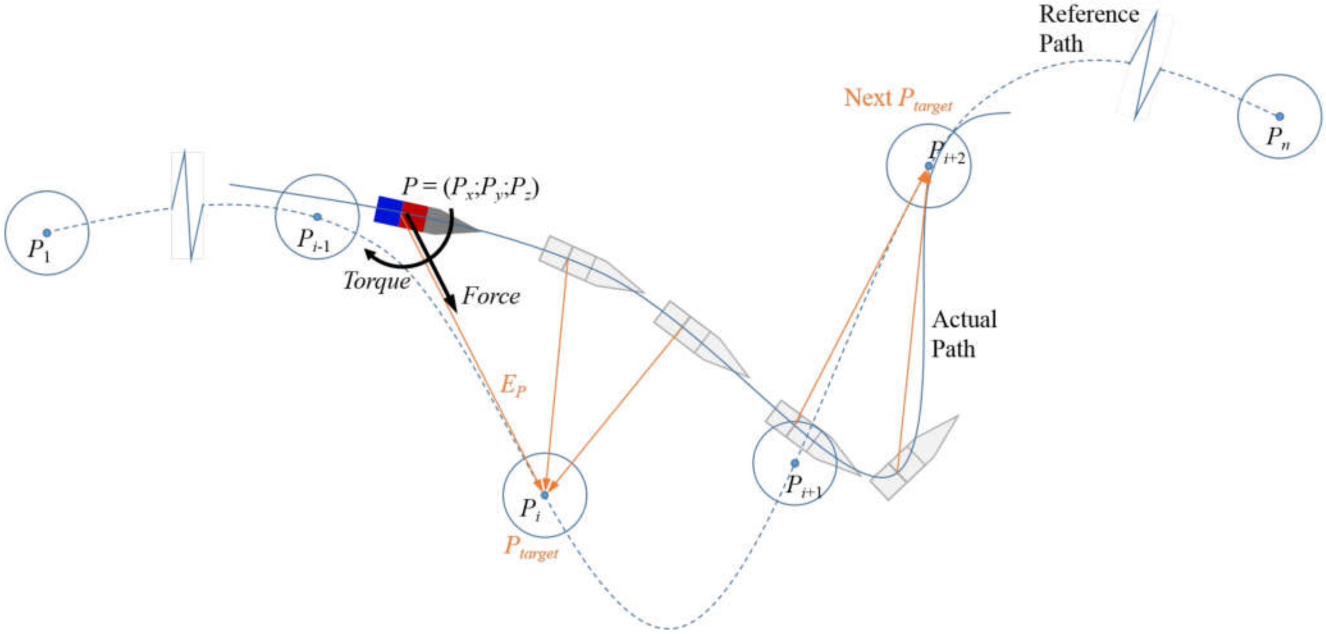

Figure S3. Illustration of the point-to-point and path following hybrid execution. P_{i+1} is reached before P_i, so the next target point is P_{i+2}; P_{i+1} is not considered as a target point for this situation.

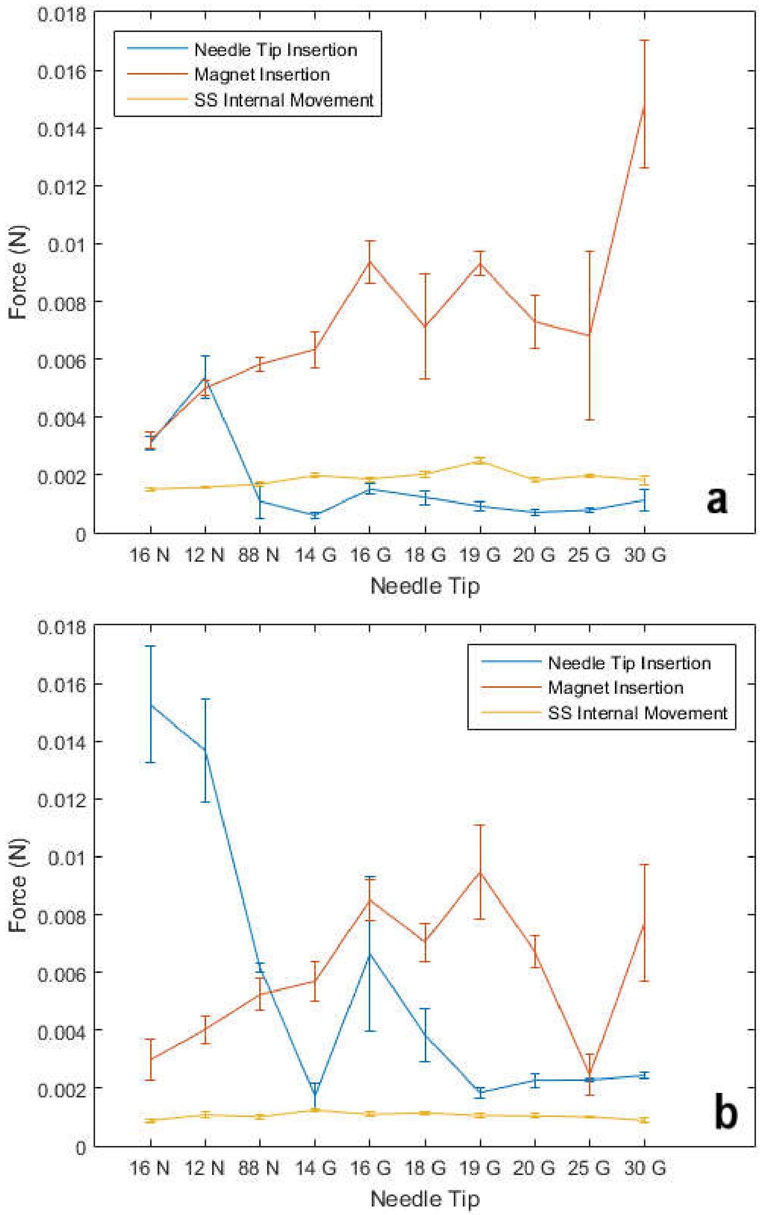

Figure S4. Comparison of the forces measured during the needle characterization tests using the setup in Fig. 4. This includes comparisons of the needle and magnet insertion forces and the steady state internal movement force for all of the 10 needles characterized in **a** stiff tissue and **b** soft tissue.

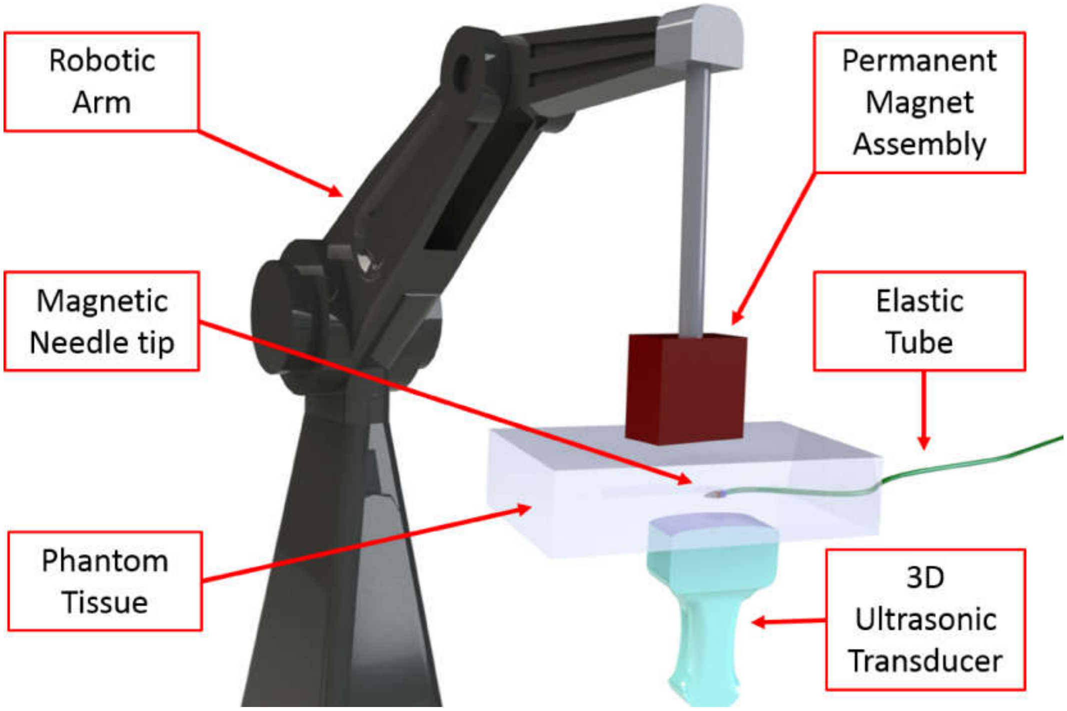

Figure S5. Visualization of potential scaled system.

Clinical prospect for needle steering.

For operations in the brain, a number of requirements must be met in order to ensure safe operations. As outlined by Hong et al., first, an entry point must be selected so that the midline of the brain is not penetrated. For cosmetic reasons, the entry point should also be selected posterior to the hairline. For path planning, all sulci should be avoided because they often have small blood vessels at the base. Ventricles and large blood vessels should also be avoided. While investigating stimulation of subthalamic nucleus, over 40 different possible paths utilizing a maximum radius of curvature of 40 mm were found, and even more paths become possible with an increasingly small radius of curvature[38].

Although needle steering has not achieved widespread clinical use, robotic needle insertion is now being implemented during clinical procedures. In August 2019, Hiraki et al. reported the first-in-human successful implementation of robotic guided straight needle insertion in ten CT fluoroscopy–guided biopsies, targeting the kidney, lung, psoas muscle, anterior mediastinum, and the adrenal gland. In this paper, the authors report that the needle insertion robot offered accuracy comparable to that done manually, with the added benefit of reduced radiation exposure to the physician. It is relevant to note that potential test subjects in whom at-risk internal structures were located within 10 mm of the planned needle path were disqualified from participating in the surgical trial[63]. Conceptually, a scaled version of the proposed magnetic steering and actuation method would be able to improve upon this qualification due to improved achievable curvature. Additionally, Hiraki et al. cite research on traditional needle steering, comparing their setup to the robotic needle steering system developed by Shahriari et al., which utilizes a traditional beveled-tip needle steering approach[64]. This recent step for robotic needle systems proves that continuing research will produce clinically useful tools.

Finally, as discussed in the introduction, the authors stress that medical professionals do in fact believe that needle steering will be a boon to clinical applications and only 2% of the respondents to the survey did not believe that steerable needles would make new interventions possible[5].

Control using the stationary electromagnetic system.

In order to develop a system for remote magnetic control, it is necessary to understand the dynamics of a magnetized body under the influence of a magnetic field and gradient.

Torque and force on a magnetized body in a magnetic field

For a magnetized body like permanent magnets, the magnetic moment is rigidly connected to the body and has a constant magnitude. We assume the needle has magnetic moment of $\mathbf{M}$ in units A.m^2.

Applied torques

By considering magnetic moment of a body at its center of mass, torque (N.m) acting on the body is as follows:

$$\mathbf{T} = \mathbf{M} \times \mathbf{B}, \tag{1}$$

where $\mathbf{B}$ is the magnetic flux density at the location of $\mathbf{M}$ in units T (Teslas). The relation between magnetic flux density and the applied magnetic field $\mathbf{H}$ in units A/m is:

$$\mathbf{B} = \mu_0 \mathbf{H}, \tag{2}$$

where $\mu_0 = 4\pi \times 10^{-7}$ T.m/A is the permeability of free space.
Applied torque tries to align the magnetic moment with the external magnetic flux density. For the bodies with a uniform magnetization, it is impossible to apply torque about the axis of $\mathbf{M}$ which limits the control to 5-DOF for such bodies. Using skew-symmetric matrix form of $\mathbf{M}$, we can write equation 1 as the following:

$$\mathbf{M} \times \mathbf{B} = \mathrm{Sk}(\mathbf{M})\mathbf{B}, \tag{3}$$

where

$$\mathrm{Sk}(\mathbf{M}) = \mathrm{Sk}\left(\begin{bmatrix} m_x \\ m_y \\ m_z \end{bmatrix}\right) = \begin{bmatrix} 0 & -m_z & m_y \\ m_z & 0 & -m_x \\ -m_y & m_x & 0 \end{bmatrix} \tag{4}$$

m_x, m_y and m_z are the components of $\mathbf{M}$ in respect to the world frame.

Applied forces

Applied force (N) acting on a magnetized body with magnetic moment of $\mathbf{M}$ is as follows:

$$\mathbf{F} = (\mathbf{M} \cdot \nabla)\mathbf{B}. \tag{5}$$

Considering the constraint of $\nabla \times \mathbf{B} = 0$ in the Maxwell equations for the zero electrical current in the magnetized body, equation 5 can be expressed as:

$$\mathbf{F} = \begin{bmatrix} \frac{\partial \mathrm{B}_x}{\partial x} & \frac{\partial \mathrm{B}_y}{\partial x} & \frac{\partial \mathrm{B}_z}{\partial x} \\ \frac{\partial \mathrm{B}_x}{\partial y} & \frac{\partial \mathrm{B}_y}{\partial y} & \frac{\partial \mathrm{B}_z}{\partial y} \\ \frac{\partial \mathrm{B}_x}{\partial z} & \frac{\partial \mathrm{B}_y}{\partial z} & \frac{\partial \mathrm{B}_z}{\partial z} \end{bmatrix} \mathbf{M} = \begin{bmatrix} \frac{\partial \mathbf{B}}{\partial x} & \frac{\partial \mathbf{B}}{\partial y} & \frac{\partial \mathbf{B}}{\partial z} \end{bmatrix}^T \mathbf{M} \tag{6}$$

Magnetic control of a magnetized body.

By having a fixed configuration of electromagnets, it is possible to compute the magnetic flux density that each electromagnet can generate throughout the workspace. For any given point $\mathbf{P}$ in the workspace, the flux density generated by the eth electromagnet carrying i_e current can be expressed by the vector $\mathbf{B}_e(\mathbf{P})$. The magnetic flux density of each electromagnet is linearly dependent to the electrical current that goes through it. By defining a unit-current magnetic flux density vector as $\tilde{\mathbf{B}}_e(\mathbf{P})$ with units of T/A. By measuring the electrical current and having the unit-current magnetic flux density vector of the eth electromagnet, its magnetic flux density can be calculated using the following relation:

$$\mathbf{B}_e(\mathbf{P}) = \tilde{\mathbf{B}}_e(\mathbf{P}) i_e. \tag{7}$$

By staying in linear magnetization region of the electromagnetic coils we can assume that the magnetic flux density at a given point in the workspace is a linear summation of each electromagnet's magnetic flux density:

$$\mathbf{B}(\mathbf{P}) = \sum_{e=1}^{n} \mathbf{B}_e(\mathbf{P}) = \sum_{e=1}^{n} \tilde{\mathbf{B}}_e(\mathbf{P}) i_e. \tag{8}$$

Equation (8) can be expressed in matrix form:

$$\mathbf{B}(\mathbf{P}) = \begin{bmatrix} \tilde{\mathbf{B}}_1(\mathbf{P}) & \dots & \tilde{\mathbf{B}}_n(\mathbf{P}) \end{bmatrix} \begin{bmatrix} i_1 \\ \vdots \\ i_n \end{bmatrix} = \beta(\mathbf{P}) I \tag{9}$$

where I is the $n \times 1$ array containing the current for each electromagnetic coil and $\beta(\mathbf{P})$ is a $3 \times n$ matrix containing the unit-current flux density of each coil in point $\mathbf{P}$. For generating the matrix $\beta(\mathbf{P})$, the unit-current magnetic flux densities were measured on a grid of points in the workspace and then for any given point the matrix was obtained by interpolation.
Similar to magnetic flux density, the derivative of the magnetic flux density for a given position $\mathbf{P}$ in the fixed frame of workspace can be expressed as follows:

$$\begin{aligned} \frac{\partial \mathbf{B}(\mathbf{P})}{\partial x} &= \begin{bmatrix} \frac{\partial \tilde{\mathbf{B}}_1(\mathbf{P})}{\partial x} & \dots & \frac{\partial \tilde{\mathbf{B}}_n(\mathbf{P})}{\partial x} \end{bmatrix} \begin{bmatrix} i_1 \\ \vdots \\ i_n \end{bmatrix} = \beta_x(\mathbf{P}) I \\ \frac{\partial \mathbf{B}(\mathbf{P})}{\partial y} &= \begin{bmatrix} \frac{\partial \tilde{\mathbf{B}}_1(\mathbf{P})}{\partial y} & \dots & \frac{\partial \tilde{\mathbf{B}}_n(\mathbf{P})}{\partial y} \end{bmatrix} \begin{bmatrix} i_1 \\ \vdots \\ i_n \end{bmatrix} = \beta_y(\mathbf{P}) I, \\ \frac{\partial \mathbf{B}(\mathbf{P})}{\partial z} &= \begin{bmatrix} \frac{\partial \tilde{\mathbf{B}}_1(\mathbf{P})}{\partial z} & \dots & \frac{\partial \tilde{\mathbf{B}}_n(\mathbf{P})}{\partial z} \end{bmatrix} \begin{bmatrix} i_1 \\ \vdots \\ i_n \end{bmatrix} = \beta_z(\mathbf{P}) I \end{aligned} \tag{10}$$

Similar to the magnetic flux density, $3 \times n$ matrices for unit-current gradient ($\beta_x(\mathbf{P})$, $\beta_y(\mathbf{P})$ and $\beta_z(\mathbf{P})$) can be calculated using an interpolation between the pre-obtained unit-current gradient on a grid of points. Using equations 1 and 5 Magnetic torque and force on a magnetized body at point $\mathbf{P}$ can be expressed as:

$$\begin{bmatrix} \mathbf{T} \\ \mathbf{F} \end{bmatrix} = \begin{bmatrix} \mathrm{Sk}(\mathbf{M})\beta(\mathbf{P}) \\ \mathbf{M}^T \beta_x(\mathbf{P}) \\ \mathbf{M}^T \beta_y(\mathbf{P}) \\ \mathbf{M}^T \beta_z(\mathbf{P}) \end{bmatrix} \begin{bmatrix} i_1 \\ \vdots \\ i_n \end{bmatrix} = A_{T,F}(\mathbf{M}, \mathbf{P}) I \tag{11}$$

$A_{T,F}(\mathbf{M},\mathbf{P})$ is a $6 \times n$ matrix relates the electrical currents of n electromagnets to the torque and force applied at the point $\mathbf{P}$ on a magnetized body of $\mathbf{M}$ magnetization. To find the required currents in electromagnets to achieve a desired set of torque and force, pseudoinverse of $A_{T,F}(\mathbf{M},\mathbf{P})$ can be used:

$$I = A_{T,F}(\mathbf{M},\mathbf{P})^{\dagger} \begin{bmatrix} \mathbf{T}_{desire} \\ \mathbf{F}_{desire} \end{bmatrix} \tag{12}$$

To find the pseudoinverse of $A_{T,F}(\mathbf{M},\mathbf{P})$, singular value decomposition is used. $A = U\Sigma V^T$, where Σ is the $6 \times n$ the singular value matrix, U is the 6×6 orthonormal matrix which its columns are output singular vectors, and V is the $n \times n$ orthonormal matrix which columns are input singular vectors. Using this method the pseudoinverse will be $A^{\dagger} = V\Sigma^{\dagger}U^T$.

Magnetic control of magnetized body in fluids.

In fluids, a magnetized body gets aligned with the magnetic flux density since there is not much resistance from the environment on it's rotation. In such cases, the control can be modified and instead of torque, magnetic flux density can be used for defining the desired orientation:

$$\begin{bmatrix} \mathbf{B} \\ \mathbf{F} \end{bmatrix} = \begin{bmatrix} \beta(\mathbf{P}) \\ \mathbf{M}^T \beta_x(\mathbf{P}) \\ \mathbf{M}^T \beta_y(\mathbf{P}) \\ \mathbf{M}^T \beta_z(\mathbf{P}) \end{bmatrix} \begin{bmatrix} i_1 \\ \vdots \\ i_n \end{bmatrix} = A_{B,F}(\mathbf{M},\mathbf{P})I \tag{13}$$

And then the current is calculated as:

$$I = A_{B,F}(\mathbf{M},\mathbf{P})^{\dagger} \begin{bmatrix} \mathbf{B}_{desire} \\ \mathbf{F}_{desire} \end{bmatrix} \tag{14}$$
